# Macrophages and Mast Cells in the Gastric Mucosa of Patients with Obesity Undergoing Sleeve Gastrectomy

**DOI:** 10.3390/jcm13154434

**Published:** 2024-07-29

**Authors:** Michele Ammendola, Francesca Vescio, Cataldo Rotondo, Franco Arturi, Maria Luposella, Valeria Zuccalà, Caterina Battaglia, Domenico Laganà, Girolamo Ranieri, Giuseppe Navarra, Silvia Curcio, Viviana Danese, Lucia Franzoso, Giuseppe Massimiliano De Luca, Francesco Paolo Prete, Mario Testini, Giuseppe Currò

**Affiliations:** 1 Science of Health Department, Digestive Surgery Unit, University “Magna Graecia” Medical School, University Hospital “R. Dulbecco”, 88100 Catanzaro, Italy; michele.ammendola@unicz.it (M.A.); vescio.francesca@gmail.com (F.V.); crotondo63@gmail.com (C.R.); silvia-curcio@libero.it (S.C.); 2 Department of Medical and Surgical Sciences, University “Magna Graecia” Medical School, Internal Medicine Unit, Outpatient Unit for the Treatment of Obesity, University Hospital “R. Dulbecco”, 88100 Catanzaro, Italy; arturi@unicz.it; 3 Cardiovascular Disease Unit, General Hospital, 88100 Catanzaro, Italy; marilyn_luposella@live.it; 4 Pathology Unit, “R. Dulbecco” University Hospital, 88100 Catanzaro, Italy; valezy@libero.it; 5 Department of Experimental and Clinical Medicine, “Magna Graecia” University Medical School, Radiology Unit, “R. Dulbecco” University Hospital, 88100 Catanzaro, Italy; dtbattagliacat@gmail.com (C.B.); domenico.lagana@unicz.it (D.L.); 6 Oncology Unit, IRCCS National Cancer Istitute “Giovanni Paolo II”, 70100 Bari, Italy; giroran@tiscalinet.it; 7 Department of Human Pathology of Adult and Evolutive Age, Surgical Oncology Division, “G. Martino” Hospital, University of Messina, 98121 Messina, Italy; gnavarra@unime.it; 8 Department of Chemistry and Technologies Pharmaceuticals, University of Bari “Aldo Moro”, 70100 Bari, Italy; danesevd@gmail.com; 9 Department of Anesthesia and Intensive Care, “SS. Annunziata Hospital”, 74121 Taranto, Italy; franzosolucia@yahoo.it; 10 Academic Unit of General Surgery “V. Bonomo”, Department of Precision and Regenerative Medicine and Ionian Area, University of Bari “A. Moro”, 70100 Bari, Italy; pretef@gmail.com (F.P.P.); mario.testini@uniba.it (M.T.); 11 Science of Health Department, University “Magna Graecia” Medical School, “R.Dulbecco” University Hospital, General Surgery Unit, 88100 Catanzaro, Italy; currog@unicz.it

**Keywords:** obesity, microenvironment, sleeve gastrectomy, immune cell, cancer, inflammation

## Abstract

**Background.** Adipose tissue macrophages (ATMs) and mast cells (MCs) play a role in immune responses. More recently, their involvement in tumor angiogenesis and chronic inflammatory conditions in patients with obesity has been discovered. Furthermore, a higher BMI (Body Mass Index) value corresponds to a higher inflammatory state. In particular, gastric tissue in obesity (GTO) is characterized by Macrophages, Mast Cells Positive to Triptase (MCPT), and neo-formed microvessels (MVD). **Materials and Methods.** We collected gastric tissue samples from December 2021 to December 2022. The patients selected had a BMI > 35 kg/m^2^ with different comorbidities. Regarding the surgery, surgeons executed a Laparoscopic Sleeve Gastrectomy (LapSG). Gastric tissue was analyzed by immunohistochemistry and morphometrical assay, comparing “obese-related” gastric tissue to normal gastric tissue. Furthermore, tissue parameters were correlated with important clinicopathological features. **Results.** We collected thirty gastric tissue samples from thirty patients with obesity. Blood tests, Electrocardiogram (ECG), esophagogastroduodenoscopy (EGDS) associated with a urea breath test, and chest X.R. were performed. A significant correlation between ATMs, MCPT, MVD, and BMI was found in GTO. Pearson *t*-test analysis was conducted (r ranged from 0.67 to 0.71; *p*-value < 0.05). **Conclusions**. These preliminary data suggest that ATMs, MCPT, and MVD related to BMI can play a role in both gastric tissue angiogenesis and inflammation inducing a tissue change that could lead to gastric inflammation or cancer diseases.

## 1. Introduction

**Figure 1:**
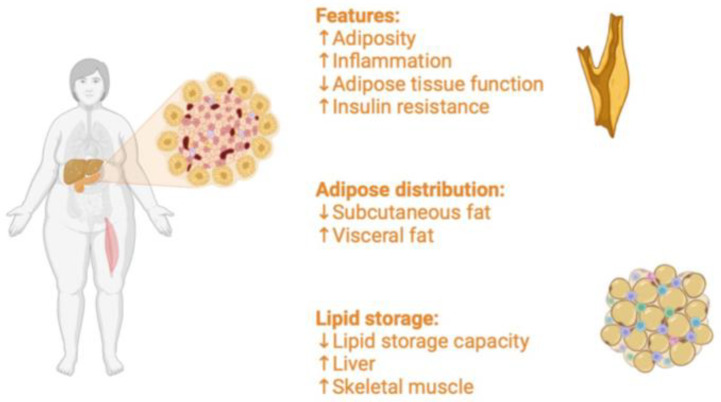
Metabolism in patients with obesity.

Obesity is a global health disease involving adults and children, primarily in occidental countries [1]. Body Mass Index (BMI) represents the value by which obesity is classified. Thus, a BMI greater than 30 kg/$m^{2}$ indicates a state of obesity. Moreover, obesity can be subclassified into four types: I (BMI between 30 and 34.9 kg/$m^{2}$), II (BMI between 35 and 39.9 kg/$m^{2}$), III (BMI ≥ 40 kg/$m^{2}$), and IV (BMI ≥ 50 kg/$m^{2}$). Obesity derives from a chronic imbalance between caloric intake and energy consumption. The most critical capacity of adipose tissue (AT) is that the adipocytes can accumulate large amounts of lipids within droplets. This leads patients with obesity to a chronic inflammatory state, causing metabolic dysfunctions and insulin resistance (IR) (Figure 1) [2]. The chronic inflammation results from high levels of adiposity. It is associated with the development of several inflammatory conditions mediated through the profound effects of adipokines on a broad range of immune cells, including dendritic cells, innate lymphoid cells, and neutrophils [3]. Albeit the molecular mechanism is still unclear, there is consensus regarding the activation of adipose tissue macrophages (ATMs), which contribute to sustaining a chronic inflammatory condition in obesity [4,5]. A specific spectrum of macrophages populates obese AT, resembling neither the M1 nor M2 type, rather a particular population characterized by metabolically activated (MMe) or oxidized (Mox) forms. These kinds of MMe and Mox macrophages are produced in response to metabolic cues like free fatty acids, high insulin, high glucose, oxidized phospholipids, and oxidized LDL. Furthermore, they express different cell-surface proteins, including ABCA 1, CD36, and PLIN2 for MMe; heme- oxygenase-1 (HO-1), sulforedoxin-1 (Srnx-1), thioredoxin-1 reductase (Txnrd-1) for Mox [6]. In obese AT, ATMs MMe and Mox species produce proinflammatory cytokines like IL-6, Il-1β, Tumor Necrosis Factor-α (TNF-α) [7], promote T-cell activation, lysosomal activity, and lipid debris scavenging.

**Figure 2:**
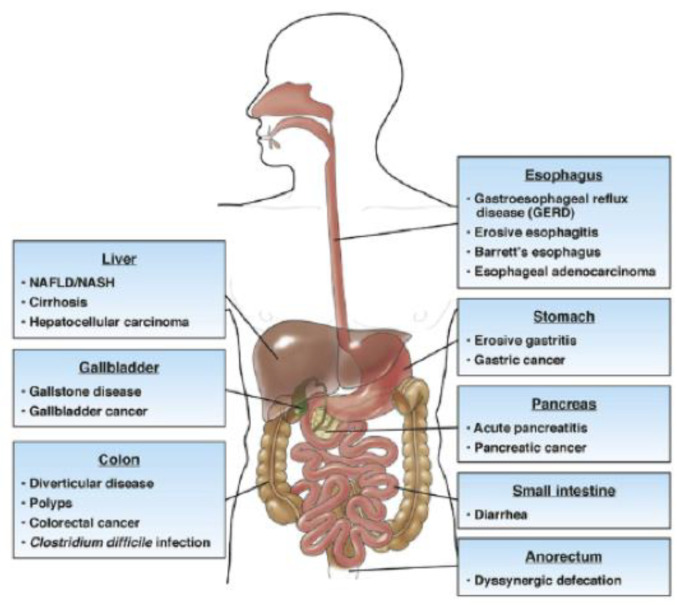
Gastrointestinal alterations in patients with obesity.

Adipose tissue is also a reservoir of mast cells whose number increases in patients with obesity. Their degranulation generates the secretion of strong proinflammatory and regulatory mediators, as well as tryptase and chymase, cytokines/chemokines. Furthermore, mast cells are strictly associated with a proinflammatory status in adipose tissue by their indirect impact on immune cell attraction and activation. They promote adipose tissue remodeling and fibrosis by adipocyte differentiation, fibroblast proliferation, and enhancing extracellular matrix protein expression [8]. Adipose tissue angiogenesis controls adipocyte metabolism and establishes communication between adipose tissue and the rest of the body. It is required also for progenitor cell proliferation and tissue remodeling. Applying single-cell and single-nuclei sequencing has opened our horizons to the role of specific signaling pathways operating between endothelial cells, adipocytes, and progenitor cells and their derangements in obesity [9]. In this way, there is an alteration of gastric motility and a consequential impact on its mucosa. Obesity has become a challenge for different medical professionals, from gastroenterologists to surgeons. Hence, it is important to underline how obesity can involve several pathological processes, representing a risk factor for developing type II diabetes, hypertension, stroke, cardiovascular disease, dyslipidemia, osteoarthritis, and asthma. The surgical procedures for obesity have grown since the mid-twentieth century, reducing BMI and comorbidities, particularly type II diabetes and hypertension [10]. Obesity also modifies the gastrointestinal tract (Figure 2) and is a risk factor for gastric cancer and cardiac tumors [11]. However, no substantial evidence exhibits the cellular modifications in the gastric mucosa of patients with obesity. Throughout this study, we have found specific inflammatory cell populations in the gastric mucosa of selected patients with obesity. 

## 2. Material and Methods

### 2.1. Patient Sampling

This study was conducted in the period between December 2021 and December 2022 at the Digestive Surgery Unit of the University Hospital “R.Dulbecco”, “Magna Graecia” of Catanzaro (Italy). We collected thirty gastric tissue samples in patients with obesity and in thin and healthy patients who did a biopsy for the control of malignant or benign pathologies. Blood tests, ECG, EGDS associated with a urea breath test, and chest X.R. were performed; the patients’ characteristics are summarized in Table 1. As reported in Table 1, more than half were female (70%). Diabetes Mellitus type 2 and OSAS represent the more essential comorbidities. Furthermore, *H. pylori* infection was not detected by definitive histological examination. The overall stay length was five days. In addition, every patient was analyzed by a multidisciplinary team consisting of a nutritionist, psychiatrist, endocrinologist, radiologist, anesthetist, and surgeon. The endocrinologist video helped avoid secondary obesity derived from Cushing’s disease, PCOS, or other obesity-related diseases. The Psychosocial Predictors Questionnaire and Environmental Predictors Questionnaire were submitted to exclude psychiatric illness (binge eating disorder, bulimia nervosa, depression) and confirm the surgery as a therapeutic option. The patients selected had a BMI > 35 kg/m^2^ with different comorbidities. Regarding the surgery, surgeons executed a Laparoscopic Sleeve Gastrectomy (LapSG), and all patients, based on the Helsinki Declaration, gave informed consent. This study was approved by the Ethics Committee of the “R.Dulbecco” Hospital, “Magna Graecia” University, Catanzaro. Gastric tissue was analyzed by immunohistochemistry and morphometrical assay, comparing “obese-related” gastric tissue to normal gastric tissue. 

### 2.2. Immunohistochemistry

ATMs, Mast Cells Positive to Tryptase (MCPT), and Micro-Vascular Density (MVD) were detected through immunohistochemistry using a three-layer biotin-avidin-peroxidase system [12]. Six μm thick serial sections of formalin-fixed and paraffin-embedded gastric tissue in obesity (GTO) and in case–control normal tissue (NT) were cut. Obtained slides were processed with a microwave oven at 500 W for 10 min, and then, the endogenous peroxidase enzyme was inhibited with 3% hydrogen peroxide solution. Subsequently, slides were stained with the following primary antibodies: anti-tryptase (clone AA1; Dako, Glostrup, Denmark) diluted 1:100 for 1 h at room temperature for MCs identification, anti-CD68 (clone KP1; Dako, Glostrup, Denmark) diluted 1:100 for 1 h at room temperature ATM identification, anti-CD31 antibody (QB-END 10; Bio-Optica Milan, Milan, Italy) diluted 1:50 for 1 h at room temperature as a pan-endothelial marker. The immunoreactivity was highlighted by employing a biotinylated secondary antibody, an avidin-biotin-peroxidase complex red chromogen (LPS, K0640, Dako, Glostrup, Denmark). Cell nuclei were stained utilizing Gill’s hematoxylin no. 2 (Polysciences, Warrington, PA, USA). No primary antibody was employed in negative controls.

### 2.3. Morphometrical Assay

**Figure 3:**
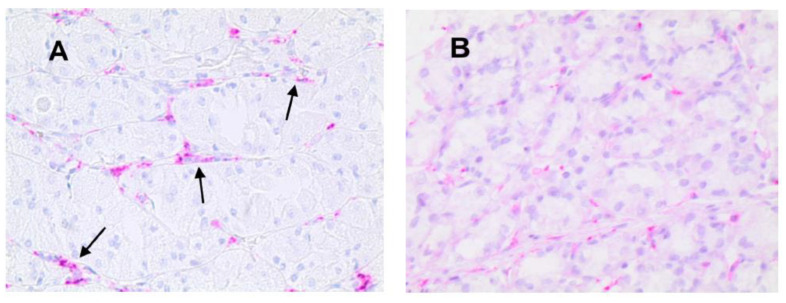
(**A**) Gastric tissue in obesity and red-stained macrophages to the anti-CD68 antibody. Arrows indicate single macrophages near microvessels (×20 magnification). (**B**) Case–control in normal tissue.

**Figure 4:**
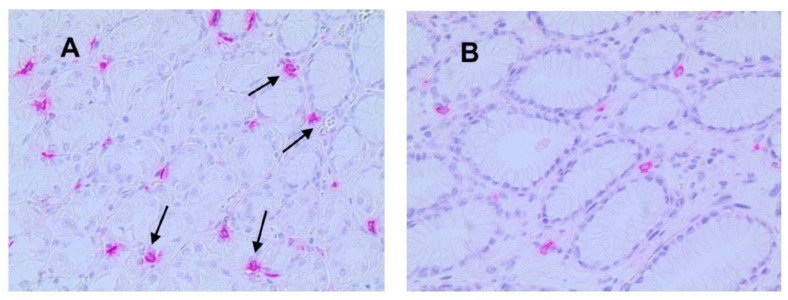
(**A**) Gastric tissue in obesity and red-stained mast cells positive to the anti-tryptase antibody. Arrows indicate single mast cells near microvessels (×20 magnification). (**B**) Case–control in normal tissue.

**Figure 5:**
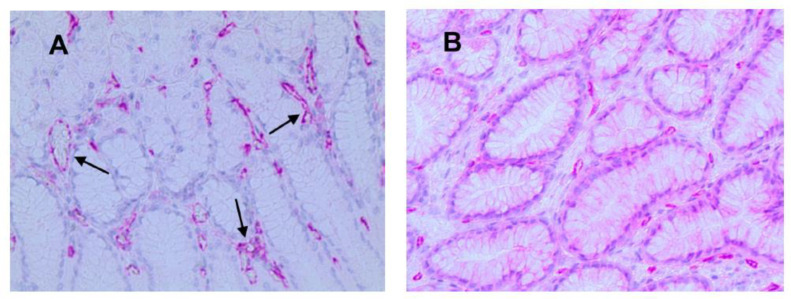
(**A**) Gastric tissue in obesity and red-stained microvessels positive to anti-CD31 antibody. Arrows indicate single microvessels with a visible lumen (×20 magnification). (**B**) Case–control in normal tissue.

Light microscopy integrated with an image analysis system (AXIO, Scope A1, ZEISS, Oberkochen, Germany) was utilized. For each serial section of GTO and NT, the five most immunostained areas (hot spots) were selected at low magnification. Subsequently, ATMs, MCPT, and MVD were assessed at ×20 magnification (0.19 mm^2^ area) in the five identified hot spots for each serial section (Figure 3, Figure 4 and Figure 5). 

### 2.4. Statistical Analysis

**Figure 6:**
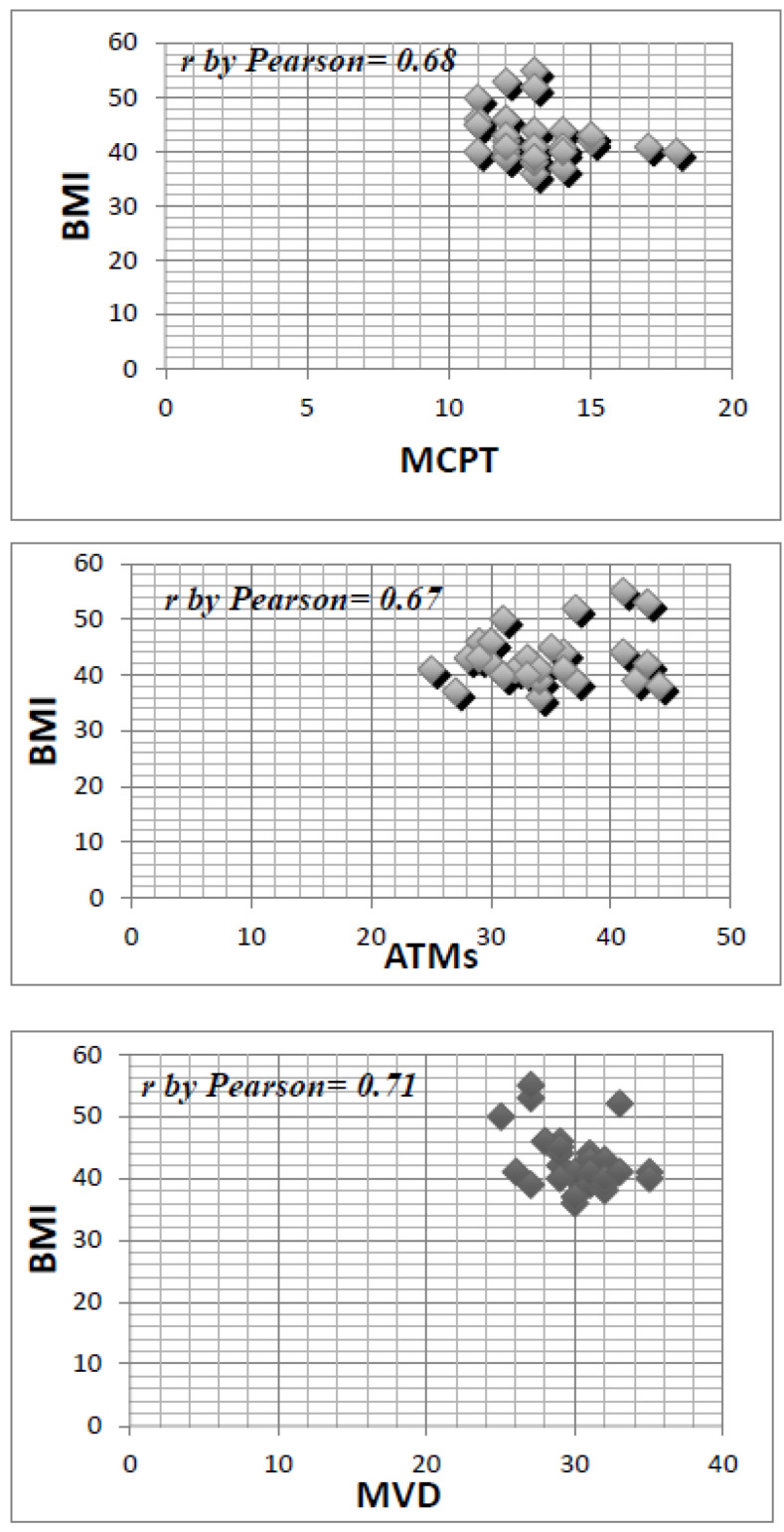
Pearson’s (*r*) correlation analysis between ATMs, MCPT, MVDm and BMI (Body Mass Index).

The mean value for each section and in the global series was obtained for all the analyzed parameters in both GTO and NT groups. Student’s *t*-test measured the difference between groups. Mean values ± 1 standard deviation (S.D.) of all the evaluated tissue parameters are reported in Table 2. Correlations between ATMs, MCPT, MVD, and BMI were calculated using Pearson’s (*r*) analysis (Figure 6). Correlations among all the analyzed parameters and the main clinical-pathological features listed in Table 2 were performed by the Chi-square test (χ^2^). All analyses were considered statistically significant with a *p* < 0.05. Statistical analysis elaboration was performed with the SPSS v28 statistical software package (SPSS, Inc., Chicago, IL, USA).

## 3. Results

### 3.1. Immunohistochemical Analysis

In GTO, microvessels, macrophages, and mast cells have a numerically greater expression with non-homogeneous and irregular distribution in the area compared to control cases. Additionally, as indicated by the arrows, the expression of macrophages and mast cells is almost contiguous to microvessels, a sign of angiogenesis and cell proliferation stimulus (Figure 3, Figure 4 and Figure 5). 

### 3.2. Statistical Analysis

The mean value ± standard deviation (S.D.) regarding ATMs, MCPT, and MVD in NT was 14 ± 5, 6 ^a^ ± 4, and 13 ± 5 ^a^, respectively, and the mean value ± S.D. in GTO was 38 ± 9, 13 ^a^ ± 6, and 30 ± 7 ^a^, respectively (Table 2). Differences in terms of mean value ± S.D. between NT and GTO were significant for each analyzed tissue biomarker (*p* < 0.05). Data demonstrated that ATMs, MCPT, and MVD significantly increased from NT to GTO. Statistical evaluation by Pearson analysis in GTO showed a significant correlation between MCPT and BMI (r = 0.68), ATMs and BMI (r = 0.67), and MVD and BMI (r = 0.71) (Figure 6). Any other correlation among ATMs, MCPT, MVD, BMI, and the main clinical pathological characteristic was found.

## 4. Discussion

The current study was a retrospective analysis of thirty samples of gastric mucosa collected after LapSG. From immunochemistry results, it was demonstrated that typical macrophages occupy obese gastric mucosa due to chronic inflammation, typical in obese AT, provoking an autocrine and paracrine loop, which influences the gastric cellular population. In the gastric mucosa, this proinflammatory microenvironment recruits mast cells and helps microvessel formation and, consequently, a neoangiogenesis process. For instance, some authors, like Csendes and Dutta [13,14], investigated 426 and 101 patients with morbidity and obesity with endoscopic biopsies, which enlightened the presence of gastritis in more than half of the samples. Moreover, they associated a higher BMI with erosive gastritis, underlying the relationship between the deposit of fat visceral tissue and a chronic gastric inflammatory state. Delgado-Aros et al. have already demonstrated the association between higher BMI, higher fasting gastric volume, and a reduced sense of satiation [15,16]. Chronic gastric inflammation in patients with obesity is due to several factors like tumor necrosis factor-α (TNF-α), leptin, and adiponectin. For instance, low serum levels of adiponectin detected in obese subjects demonstrate, compared to normal subjects, the presence of erosive gastritis [17,18]. Hence, it indicates that adiponectin systematically plays an anti-atherogenic, anti-diabetic, and anti-inflammatory role. Gastric mucosa can be affected by inflammation in an active or inactive state. As we have already reported, typical macrophages found were represented by MMe and Mox macrophages, which increase in AT, creating a specific microenvironment and realizing proinflammatory cytokines like TNF-α, IL-1, and IL-6. On the other hand, macrophages appear as multinuclear cells like Langherans cells, observed in granuloma during the inflammatory process [19,20]. Throughout our study, we have observed that GTO is characterized by macrophages, MCPT, and neo-formed microvessels. This kind of cellular population suggests a chronic state of inflammation in obesity. Furthermore, we have evidenced a straight correlation between BMI value and gastric mucosa state: a higher BMI corresponds to a higher inflammatory state [21]. Even though the gastric specimen detected was always the more significant curve, the macrophage population occurs in all gastric mucosa, describing a general inflammatory state that maintains an activated autocrine loop. Although macrophage polarization (MMe/Mox type) has been studied regarding its effects on the progression of obesity and obesity-associated cancer [22,23], the main mechanisms could be enhanced. In particular, it is demonstrated that adipose tissue (AT) is considered an endocrine organ to secrete adipokines and cytokines. Adipokines are involved in various metabolic and physiological signaling cascades and regulate insulin signaling, glucose uptake, fatty acid oxidation, and other metabolic processes. Cytokines, pro- and anti-angiogenetic factors, regulate inflammation and the resolution of inflammation. Obesity causes a phenotypic switch of AT, which is characterized by the appearance of inflamed, dysfunctional adipocytes along with the infiltration of immune cells in the stromal microenvironment. These inflamed adipocytes secrete proinflammatory cytokines, which, in turn, disrupt the normal function of AT itself and also contribute to the dysfunction of other types of organs. AT at this point can be considered an immune and secretory organ, and obesity is an inflammatory immune disease [24]. Eguchi et al. reported that saturated fatty acids induced beta cells to produce chemokines that attracted CD11b+ Ly-6C+ M1-type proinflammatory monocytes/macrophages to the islets [25]. In a review, Ying. et al. analyzed the role of macrophages in obesity islet inflammation explaining that islet-resident macrophages underlie the inflammatory response in obesity and mechanistically participate in the β-cell hyperplasia and dysfunction that characterizes this insulin-resistant state; also, they found that during chronic inflammation, circulating monocytes infiltrate and accumulate in inflamed tissue sites [26]. In addition, the role of inflammation in cancer has been described [27,28]; however, so far, there is no evidence for the role of inflammation activation in obesity-associated cancers, although its activation may lead to insulin resistance, one of the most independent risk factors for several types of cancer [29]. Furthermore, IL-1 is under investigation as a key molecule in inflammasome activation in obesity-associated cancers [27]. Adipose tissue, along with its chronic inflammatory status, is the major driver sustaining the association between obesity and cancer. More studies are needed to better understand the mechanisms underlying the obesity–cancer crosstalk. Epidemiological and clinical data recognize the adipocyte-secreted hormone leptin as one of the most important mediators of the link between obesity and cancer. Recently, in a study by Caruso et al. [30], the role of leptin and the leptin receptor was investigated with a finding of overexpression in patients with cancer, where this adipokine stimulates proliferation, migratory and invasive potential, and stemness capabilities in multiple types of tumors. Thus, a deeper knowledge of the mechanisms linking obesity to malignancies could lead to the identification of novel biomarkers as well as therapeutic interventions that optimize the clinical management of obese patients with cancer and could impact the onset. Before drawing our conclusions, some limitations must be discussed. First, the findings may not be representative of a broader population; Second, the study has been conducted in a single center, limiting the generalizability of our findings to other populations or settings. However, most of our findings confirm data already presented in the literature. Last, the sample can be considered small. Further studies are needed.

## 5. Conclusions

By this preliminary study, our perspective has collected more cases and increased in vivo experiments than in vitro. In this way, it is probable that the molecular pathways that regulate ATM polarization, MCPT, and MVD could be known profoundly and could explain the role of obesity as a risk factor for several diseases. Moreover, the histopathological findings in gastric mucosa could represent the starting point for discovering novel infiltrating cells or biomarkers and, later, different medical strategies to control obesity and its clinical progression to comorbidity and other pathologies. 

## Figures and Tables

**Table 1:** Characteristics of the patients examined; *n* number, BMI (Body Mass Index), DMT2 (Diabetes Mellitus type 2), OSAS (Obstructive Spleen Apnoea Syndrome).

Gender *n*	Age Range	BMI	DMT2*n*	OSAS*n*	*H. pylori*
F 21	28 ys	44.3 (Mean Value)	7	10	None
M 9	24 ys	42.4 (Mean Value)	5	2	None

**Table 2:** Main clinical-pathological features; adipose tissue macrophages (ATMs), Mast Cells Positive to Tryptase (MCPT), and Micro-Vascular Density (MVD) mean ± S.D.s as a function of obesity gastric tissue (GTO), normal tissue (NT), and statistical significance of their differences by Student’s *t*-test.

Tissue Type	MVD ×20 Magnification (0.19 mm^2^ Area)	MCPT ×20 Magnification (0.19 mm^2^ Area)	ATMs ×20 Magnification (0.19 mm^2^ Area)
NT	13± 5 ^a^	6 ^a^ ± 4	14 ± 5
GTO	30 ± 7 ^a^	13 ^a^ ± 6	38 ± 9
*p*-value (*t*-test)	*p* < 0.05	*p* < 0.05	*p* < 0.05

ATMs, MCPT, and MVD mean ± SDs as a function of obesity gastric tissue (GTO), normal tissue (NT), and statistical significance of their differences by Student’s *t*-test. ^a^ Mean ± 1 standard deviation.

## Data Availability

All data generated or analyzed during this study are included in this published article and its information files.
